# Harshness Predicts Reproduction in Brazilian Municipalities and US Counties: A Life History Theory Approach

**DOI:** 10.1177/14747049261432896

**Published:** 2026-04-15

**Authors:** Vinícius Betzel Koehler, M. D. Rutherford

**Affiliations:** 1 Department of Psychology, Neuroscience & Behaviour, 3710 McMaster University, Hamilton, Canada

**Keywords:** life history theory, teen pregnancy, harshness, unpredictability, reproduction, perceived minorities

## Abstract

Psychosocial acceleration theory (PAT) posits that harsh and unpredictable ecologies during childhood can cue humans into developing earlier and more frequent reproduction. This study tested whether variables measuring harsh and unpredictable circumstances in 4,135 Brazilian municipalities and in 2,763 US counties would predict reproductive behavior 10 to 14 years later. Data was extracted from the Brazilian Census and American Community Survey samples. A secondary analysis explored whether the percentage of visible minorities (Black and Indigenous population) would also be a predictor or mediator of the same outcomes. Partial least squares structural equation modeling and multivariate linear regression were used in the analysis of Brazil and US data, respectively. Municipalities with higher rates of lack of resources, with young mothers both married or separated, and with large families with many residents per room were predictive of higher rates of teenage and young adult mothers and of young children in Brazil. Harshness predicted the percentage of young children in US counties, but the direction of this association was mixed. Some findings were contrary to PAT predictions. Divorce rates were negative predictors of reproduction in both countries. Education and employment indicators were not significant predictors of reproduction in Brazil. Higher rates of perceived minorities were not a relevant predictor in Brazil, and they were a negative predictor of the percentage of children in the United States. Findings suggest that harsh ecologies and the proportion of children in the population impact patterns of reproduction a decade later.

## Introduction

Life history theory in psychology (LHT-P; Frankenhuis & Nettle, 2020; Nettle & Frankenhuis, 2020; Sear, 2020) describes factors that inform the allocation of resources between reproduction versus body growth and maintenance (Ellis et al., 2009; Stearns, 1992; Xu et al., 2018). LHT-P originated from life history theory in evolutionary biology (LHT-E), which started describing variation between species (Del Giudice, 2009). However, the theory also accounts for within-species variation (Albaladejo-Robles et al., 2023; Malone et al., 2022; Stone et al., 2023), including humans (Dinh et al., 2022; Richardson et al., 2020; Stearns et al., 2008).

Depending on their pattern of investments in reproduction or body growth and maintenance, species or individuals can be classified as lying on a continuum from fast to slow life history strategies (LHSs; Copping & Campbell, 2015; Sear, 2020; Stearns & Rodrigues, 2020). Species or individuals on the fast LHS end favor investments in reproduction earlier in life and focus on offspring quantity, whereas those on the slow LHS end would prioritize body growth and maintenance and offspring “quality” (Ellis et al., 2009). There are critics about this continuum both in LHT-E and LHT-P, which we discuss below, but this description keeps being used in LHT-P research (e.g., Chang et al., 2019; Wang et al., 2022; Zhu & Chang, 2020).

Among LHT-P, a great deal of attention has been given to psychosocial acceleration theory (PAT), which posits that early environmental adversity can alter the onset of puberty (most studies measure time of menarche) and other developmental milestones such as sexual debut and the age of having children (Belsky et al., 1991; Ellis et al., 2003). In PAT research, harsh and unpredictable ecologies have been associated with fast LHSs (Copping & Campbell, 2015; Griskevicius et al., 2011; Webster et al., 2014). If resources are scarce or unpredictable or if there are high levels of predation, reproducing fast and creating numerous offspring may be advantageous because it takes the opportunity for passing on genes and spreading them in the environment while conditions allow. In PAT, harshness is defined as the rates of extrinsic morbidity and mortality in the environment, whereas unpredictability is defined as stochastic variation of such rates (Ellis et al., 2009).

In practice, PAT research has been using socioeconomic measures as a proxy for harsh conditions, whereas unpredictability has been typically measured with parental absence or parental transitions (i.e., changes in the presence of parental figures due to separation, divorce, or remarriage), parental employment change, and geographical moves (Belsky et al., 2012; Ellis et al., 2003; Hartman et al., 2018; Xu et al., 2018). Faster and earlier reproduction is typically measured as time of menarche (Webster et al., 2014), time of sexual debut, and age at first birth or age at marriage (Copping & Campbell, 2015; Ellis et al., 2003; Xu et al., 2018). Among the usual unpredictability measures, parental absence seems to be the best predictor of earlier reproduction, and the first 5 or 7 years of life seem to be a critical period for this cue (Ellis et al., 2003; Simpson et al., 2012; Webster et al., 2014; Xu et al., 2018).

Recently, the assumptions and even the findings of LHT-P and of PAT have been criticized. These critics include those questioning the existence of a single fast-to-slow LHS continuum among humans (Nettle & Frankenhuis, 2020) and its utility in explaining variation in adopted traits (Stearns & Rodrigues, 2020). In fact, the notion of coherent LHSs encompassing broad suites of traits has been challenged as conceptually inconsistent with LHT-E (Sear, 2020; Stearns & Rodrigues, 2020). Although LHT-E focuses on life history traits (e.g., growth, reproduction) and on the trade-offs that shape their timing and covariation, LHT-P tends to focus on the psychological and behavioral strategies by which these traits arise. This continuum has been mainly abandoned in LHT-E (Nettle & Frankenhuis, 2020), where researchers have been using formal mathematical models to make predictions that are typically focused on a limited number of traits (Frankenhuis & Nettle, 2020; Nettle & Frankenhuis, 2020). These differences have resulted in the suggestion that LHT-P should replace the notion of single “strategies” lying on a continuum by the analyses of specific trade-offs and of the environmental cues that influence such trade-offs.

Other critics hypothesize that mortality, morbidity, and resource scarcity may no longer be present in modernized environments as much as they would be present in the environment of evolutionary adaptedness (Belsky et al., 1991; Buss, 2024). As a result, these lower levels of harshness and unpredictability would not be sufficient to cue humans into a faster LHS adoption (Nolin & Ziker, 2016; Volk, 2023, 2025). This is even more likely to be the case in developed countries, where most of the studies in the field are conducted (Sear, 2020; Volk, 2023, 2025; Webster et al., 2014; Xu et al., 2018). Indeed, studies have failed to find the expected results derived from LHT-P and PAT (e.g., Nolin & Ziker, 2016; Richardson et al., 2020, 2024; Wells et al., 2019).

There are hypotheses to explain this phenomenon that are related to PAT but use a different mechanism of explanation. One of these alternative hypotheses is that genes could mediate or confound the association between harsh and unpredictable environments and earlier reproduction (Del Giudice et al., 2015; Volk, 2025). Shared genetic factors between parents and offspring could also result in a cyclical component. Low parental investment would result in harsher and more unpredictable environments early in childhood, which could be associated—due to genes, environment, or both—with faster and earlier reproduction and again lower investment in offspring. Other hypotheses focus on cultural differences or differences in access to institutional support (Wilson, 1987; Wodtke, 2013). For example, places with reduced access to high-quality education or health care could result in riskier reproductive behavior or more unplanned reproduction. These hypotheses, however, would not explain the studies that did not find the association expected in PAT, as they mostly argue that environments with scarcer resources would be associated with earlier and more frequent reproduction.

A longitudinal design would be ideal to test hypotheses between environmental conditions and a life trajectory of prioritizing investments in either reproduction or growth and maintenance, particularly considering the developmental aspect of a critical time of exposure (0–7 years of age) to the environmental predictors and the later appearance of the outcomes. When considering the measures that are typically used (e.g., socioeconomic status, family structure, and marital relationships), census and other governmental surveys would make a valuable data source for this field (Copping, 2017). Most countries conduct censuses or other surveys periodically and measure variables similar to the variables of interest in this field. Moreover, utilizing such data sources would allow for cross-country comparisons, including non-Western and developing populations, which is more representative of the majority of the human population (Henrich et al., 2010). Cross-cultural work within human behavioral ecology and evolutionary anthropology has examined life history traits across diverse populations, including non-industrialized societies (e.g., Martin et al., 2020; also see Sear, 2020). However, the use of census data to test PAT hypotheses has not been well explored. To the best of our knowledge, LHT-P research utilizing the census or surveys of entire populations has only been done in England and Wales (Copping, 2017; Copping & Campbell, 2015). Most studies predicting human LHSs are derived from surveys with convenience samples (Sear, 2020), with either adult (e.g., Wang et al., 2022) or adolescent participants (e.g., Lordelo et al., 2011). Many of the longitudinal studies exploring LHT-P questions rely on the same samples, like the Minnesota Study of Risk and Adaptation and the Study of Early Childcare and Youth Development (Young et al., 2020), the Avon Longitudinal Study of Parents and Children (e.g., Magnus et al., 2018), or the UK Millennium Cohort Study (e.g., Kelly et al., 2017). These studies are important because they address this longitudinal characteristic of LHT-P hypotheses. The samples are large and representative of their focal population. Repeated reports on the same samples, however, reduce the independent verification of the results and can also increase the risk of type I errors.

There are four aims in the studies in this article. The first is to test if an exploratory analytical method using both PAT as a theoretical frame of work and using governmental surveys could be useful in statistically predicting reproductive patterns in an entire country's population. To do this we will investigate whether variables extracted from the Brazilian Census that are akin to the constructs of harshness and unpredictability usually present in PAT literature can predict earlier reproduction 10 years later. Following PAT literature, we hypothesize that socioeconomic stressors—typically used as a proxy for harshness—and that family configuration stressors—typically used as a proxy for parental absence—will be statistically significant predictors of earlier reproduction.

This article will be using demographic variables descriptive of people residing in a given municipality but will make interpretations based on an evolutionary psychology theory, a field in which research is usually conducted with individual-level participant data. It is worth mentioning that the data used here is observational and aggregate across geographical regions. Thus, our approach focuses on prediction and detection of patterns across geographies and cross-culturally rather than causal inference. This will limit the validity or the scope of inferences we can make from the studies in the article. On the other hand, testing this theory using a whole country's population as the “sample size” is important considering how valuable cross-cultural or universal trait findings are for evolutionary psychology (Buss, 2024).

The second aim of this article is to identify which variables, among the ones selected, will predict earlier reproduction. Among potential indicators of harshness and unpredictability present in the Brazilian Census, which ones will an exploratory analysis show to be the most statistically significant and relevant predictors of our outcome variables? We have no specific hypothesis about which variables will be selected, but we aim to discuss potential explanations for variables being or not being significant predictors of early reproduction.

The third aim of this article is to test if the findings in a different country's population (the United States) and using a different data source (the American Community Survey (ACS)) would be similar to the findings observed with the Brazilian Census. We will use a more confirmatory analytical approach in this analysis. Considering the evolutionary background of PAT, we hypothesize that, in general, the results using US data will be in the same direction as they were with Brazilian data, although we expect to find some differences due to socioeconomic and cultural differences between the two countries.

The last aim of the study is to test whether a higher percentage of visible minority groups will be predictive of earlier reproduction in Brazilian municipalities and US counties. Both countries have a long history of Black and Indigenous communities facing discrimination in education, employment, health access, the justice system, and many other settings (Aliverti et al., 2023; Bleich et al., 2019; Couto & Brenck, 2024; Serchen et al., 2022; Silva et al., 2024), and the United States observes a somewhat more recent history of racism against and discrimination against Hispanics or Latinos (Canizales & Vallejo, 2021). Following PAT rationale, it is reasonable to assume that these conditions of discrimination and racism faced by visible minority groups configure as higher levels of harshness and unpredictability in life, which could alter the LHS of these populations. For example, visible minorities could experience poor conditions regarding employment, education, housing, access to health care, or more exposure to violence. Some of these measures are usually present in the census (e.g., employment or education), while others are not (e.g., access to health care or exposure to violence). Thus, a secondary question is whether the percentage of these ethnicities (i.e., understood here as an indicator of people living in particularly harsh and unpredictable ecologies) will be either predictors of earlier reproduction or mediating variables in our primary prediction. We hypothesize that municipalities and counties with higher percentages of visible minority groups will have higher percentages of reproduction indicators.

## Young Parenthood in Brazil

### Methods

#### Data Selection and Transformation

We accessed publicly available census data from 5,507 municipalities from the 2000 Census and 5,565 municipalities from the 2010 Census from Instituto Brasileiro de Geogragia e Estatística (IBGE; Brazilian Institute of Geography and Statistics). We selected a subset of variables that are publicly available online in the *Amostra—Tabulação Avançada* (“Sample—Advanced Tabulation” in free translation) at SIDRA (Sistema IBGE de Recuperação Automática—SIDRA, 2024). This difference in the number of municipalities in 2000 and 2010 is due to the redistribution of Brazilian territory into different municipalities, mainly the division of one particular municipality into two. This difference was not controlled for in the model because it represented only 1% of municipalities, and the process of matching eliminated the new municipalities present in the 2010 Census.

All variables are expressed as the percentage of the population in a given category (e.g., people with income of one minimum wage or less, people unemployed or precariously employed, children aged 0–4 years). This subset was selected based on the variables available in the census that best map onto the variables frequently measured as proxies of harshness, unpredictability, and reproductive timing in studies in PAT. Thus, we aimed to select variables that seemed indicative of socioeconomic status (harshness), parental change (unpredictability), and earlier or frequent reproduction (reproductive timing). In many cases, as is common with secondary data research (Andersen et al., 2011), the ideal variable was not available, and we had to choose proxies for that variable. The measure of menarche was a clear case of a frequent measure in PAT literature that we could not include because this is not present in census data. Variables that did not resemble frequent measures in PAT literature were ignored, even if they could be relevant to the question in other disciplines (e.g., living in a rented or owned dwelling with or without a mortgage).

We then merged 40 variables from the 2000 Census with 10 variables from the 2010 Census and used them in an initial model. These variables were initially grouped into the following predictors: *Low income and lack of resources*, *Family/house size*, *Low education*, *Youth and married with children*, *Unemployed or precariously employed*, *Children aged 0 to 9 years old*, and *Black skin color*; and outcomes: *Early reproduction* and *Youth married.* Table 1 includes the variables that were used in the two models in the first study, and SM_Table 1 in the Supplemental materials includes the initial 40 variables extracted from the census. Both tables describe the concept the variables were intended to measure (harshness, unpredictability, or reproduction), the usual measure of such concepts in LHT-P research, the factors the variables were loaded into, the variables’ names as present in the census (freely translated from Portuguese), and the abbreviations used in this study. Refer to SM_Variables file in the Supplemental materials for a compilation of the raw data collected and how they were initially categorized.

**Table 1.** Variables in the Brazilian Model and Inferred Concepts.

LHT Concept	Usual Measure in LHT	Factor in the Model	Variables Present in the Census	Variable Abbreviation^a^
Harshness	SES measures	Low income and lack of resources	Lack of recycling of garbage collection	No recycling service
			Lack of electrical power service^b^	No electrical power
			People with income of 1 minimum wage or less	1 minimum wage or less
		Family/house size (lack of resources)	Number of people in the family—6 people	Families with 6 people
			Resident density per bedroom—more then 2 up to 3 residents	2–3 residents/bedroom
			Resident density per bedroom—more than 3 residents^c^	>3 residents/bedroom
			Number of rooms—2 rooms^c^	2 rooms in the house
Unpredictability	Parental transitions	Youth and married with children (parental transitions + having a young mother)	Divorced^c^	Separated
			Judicially separated^c^	Divorced
			Aged 15–19 living with spouse or partner	15–19 years old living w. partner
			Women aged 15–19 with children^c^	Mothers 15 to 19 years old
			Women Aged 20–24 with Children	Mothers 20 to 24 years old
−	−	Percentage of children^d^	Age group 0–4	
			Age group 5–9	
−	−	Skin color^d^	Color or ethnicity—Black	
Reproduction	Menarche, number of partners, number of children	Early reproduction	Age group 0–4	Children 0 to 4 years old
			Age group 5–9	Children 5 to 9 years old
			Women aged 15–19 with children	Mothers 15 to 19 years old
			Women aged 20–24 with children	Mothers 20 to 24 years old

*Note.* Harshness and unpredictability variables collected from census in 2000 and reproduction variables collected from 2010. LHT: Life history theory; SES: socioeconomic status.

a Variable abbreviation if retained in the final model.

b Log transformation applied to the variables.

c Square root transformation applied to the variables.

d Included in first iteration of the model but planned to be used in model comparisons.

There are two groups of variables that are not common measures of harshness and unpredictability that are worth discussing here. These variables were used as both predictors and outcomes, although with the time difference, and composed the factors *Youth and married with children* and *Early reproduction*. The first group was the percentage of children aged 0–9 years. We planned to use the percentage of children as a predictor because PAT proposes that there is a critical period in which environmental conditions cue reproductive development and as an outcome because they can indicate more frequent reproduction in that municipality. We have also planned to divide the data based on this variable to double-test this critical period for reproductive development (see the *Models* section). The second group of variables was the percentage of young people living with partners and the percentage of young mothers. They were included because of the discussion of a cyclical component—having low parental investment during childhood would cause a harsh and unpredictable childhood, leading to later low investment in offspring—in LHS adoption. Finally, these variables were also useful to assess whether associations are likely to be describing PAT's developmental phenomenon or merely geographical association. We discuss this in detail in the Discussion section.

We also used usual statistical treatments related to missing data, outliers, and data transformation. A cutoff criterion for missing values of 5% was established: any variable with 5% or more of missing values would be removed from the model. Four of the outcome variables were removed for this reason (women aged 10–14 with children; aged 10–14 living with spouse or partner; aged 10–14 married but not living together; and aged 20–24 married but not living together). None of the predictors were above the cut-off criterion. Municipalities that had any variables more than three *z-*scores apart from the mean value were removed. These were done to reduce the likelihood of patterns in missing data or outlier cases driving an effect.

Lastly, the square root and log transformations were applied to all variables. For each measure, we then selected the one (i.e., original, square root-, or log-transformed) whose distribution was closest to normality based on visual inspection of histograms and boxplots. Log transformation of two *Unemployed or precariously employed* variables transformed cases to infinity, which were then replaced by not available (NA) values and ended up being above the 5% cut-off criterion. *Unemployed or precariously employed* variables were tested but not included in the model (see more details in the Results section). After merging and *z*-score removal, 4,135 municipalities remained to be used in analysis.

#### Partial Least Squares Structural Equation Modeling

Structural equation modeling is a second-generation statistical technique, sitting between analysis of variance or multiple regressions and machine learning (Hair et al., 2022). Structural equation modeling combines factor analysis and path analysis (i.e., a series of multiple regression analyses) to examine relationships between observed and latent variables (Hair et al., 2022; Kline, 2016). It has the advantage of describing complex relationships between several variables in a single model. It has been utilized in social sciences, business, and psychology to evaluate hypothesized causal relationships and make predictions of an outcome variable that is usually a construct that cannot be directly measured (Hair et al., 2022; Kline, 2016; Maruyama, 1997).

Covariance-based structural equation modeling (CB-SEM) is the most widely used, and it is primarily a confirmatory approach (Hair et al., 2022; Kline, 2016). Partial least squares structural equation modeling (PLS-SEM), on the other hand, is primarily an exploratory approach (Hair et al., 2022). Although CB-SEM relies on fit indices by comparing the covariance matrix implied in the model with the covariance matrix found in the data (Kline, 2016), PLS-SEM relies on several statistics for the evaluation of its measurement (factor analysis) and its structural (path analysis) model (Hair et al., 2022).

PLS-SEM is a nonparametric analysis, and it is more robust than CB-SEM with formative latent variables (i.e., where items are understood to be forming the latent variable instead of the latent variable being the assumed common cause for the items), which is true for all of our predictors. It also allows for single-item measures to be included in the structural model. PLS-SEM performs similarly to CB-SEM in a wide range of cases, especially with larger sample sizes or for simple mediation models (Hair et al., 2022; Willaby et al., 2015). All of the above contribute to PLS-SEM, as opposed to CB-SEM, being a better analytical approach for this study.

However, we acknowledge that PLS-SEM has been criticized as being merely a weighting system in its measurement model; for lacking theoretical bases for model fit, which CB-SEM has, therefore not allowing for overidentification tests; and for issues with its significance tests (Rönkkö et al., 2015). We are mindful of these limitations and of the exploratory nature of this research when interpreting our findings. All analyses were conducted in R using the seminr package (Ray et al., 2018).

**Figure 1.**
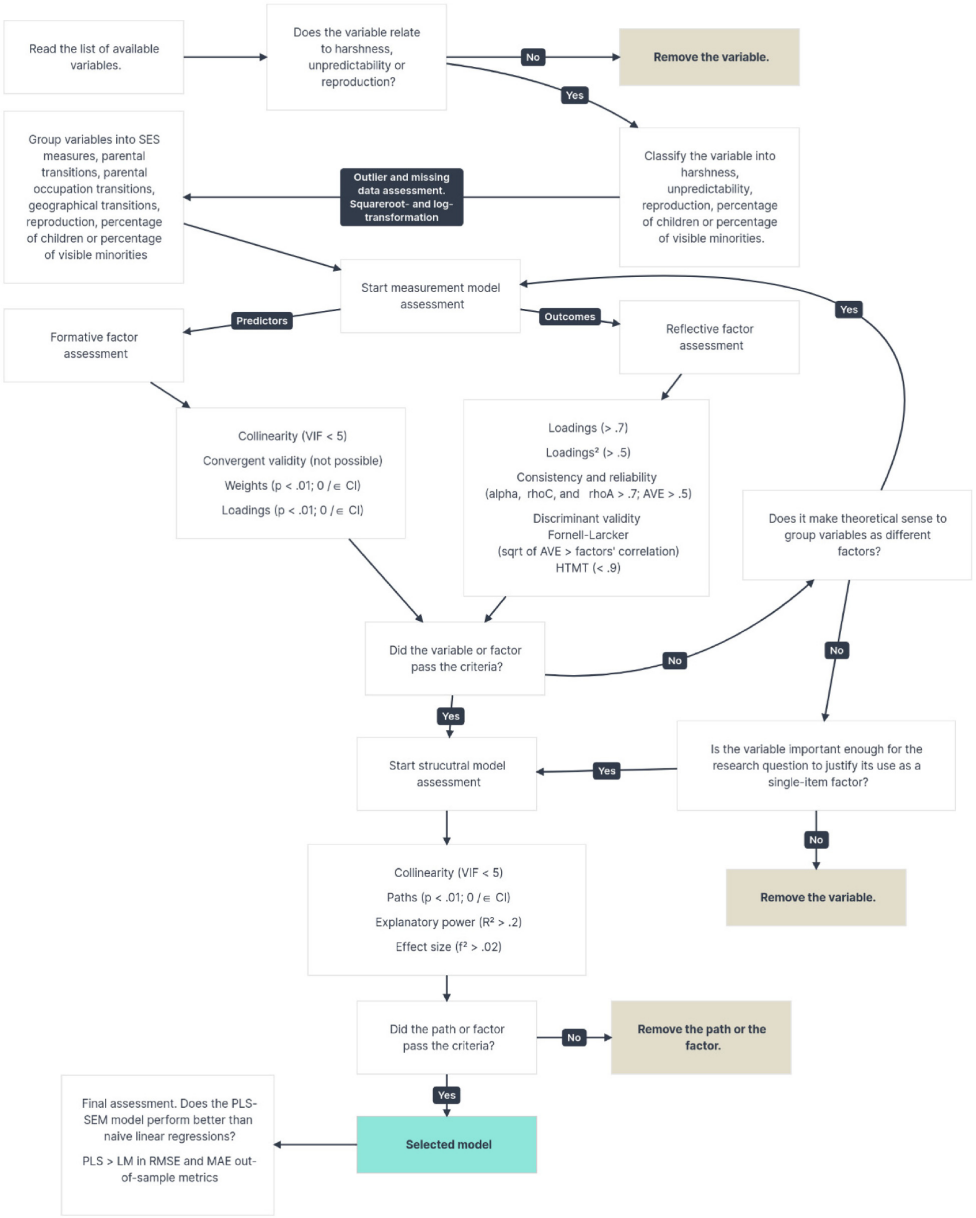
Decision-making process in the PLS-SEM analysis.

*Note.* AVE: average variance extracted; HTMT: heterotrait–monotrait ratio; PLS-SEM: partial least squares structural equation modeling.

Considering the large sample size, it would be very likely that we would find significant *P*-values in nearly all the evaluation statistics. In addition, considering that the census is the description of virtually the whole population in a given country, *P*-values are not very informative. Regardless of statistical significance, the associations found with this data will be the description of an association found in a country's population. Due to that and to the criticisms of PLS-SEM analysis, we established statistical significance of α ≤ .01 for our analysis but also examined whether confidence intervals (CIs) included zero—*which* results in a null path or loading null—and on thresholds recommended by Hair et al. (2021) for accepting the measurement and structural models. We used the following thresholds to characterize explanatory power: Adj. *R*^2^ < 0.2 = negligible; from 0.2 to 0.5 = weak; from 0.5 to 0.7 = moderate; above 0.7 = strong (Nau, 2020). The threshold for paths to be considered relevant and to be included in the model was *β* > |.1| because low *β* are at risk of the CI crossing 0, rendering the path null. Figure 1 describes the decision-making process in this analysis, including the guidelines and measures of model quality recommended by Hair et al. (2019, 2021). One exception was the absence of a convergent validity analysis for formative factors. This has been acknowledged before as an issue with research using secondary data (Hair et al., 2019).

#### Models

Predictors were set as formative latent variables (i.e., a group of items that sufficiently describe a factor), whereas outcomes were set as reflective latent variables (i.e., when the group of items are understood to be caused by the factor). We argue that our predictors are not reflective latent variables because constructs such as people living on minimum wage or less and lacking access to public services are caused by the greater latent construct of *Lack of resources*. They are, in fact, formative latent variables because we are using PAT theory, assessing these variables, and assuming that they sufficiently (or reasonably, at least) describe people living with a *lack of resources*. The same was assumed to be true for the other predictors. However, our outcomes were assumed to be reflective. PAT states that early environmental conditions cue individuals into adopting different LHSs. The different LHSs can be assumed to be the common cause for the outcome latent variables.

Two model comparisons were conducted. One tested whether the percentage of visible minorities in Brazilian municipalities would be a statistically significant and relevant predictor of early reproduction or a mediator between harsh conditions and early reproduction. The other model used the selected model. The selected model, however, was tested with the subset of top and bottom quartiles of the sample based on the percentage of children 0–4 years old and 5–9 years old. In the first model comparison, we hypothesized that ethnicity would be a statistically significant predictor and/or mediator. In the second model comparison, we hypothesized that the paths and explanatory power of the model with top quartiles’ subset would be higher than the paths and explanatory power of the bottom quartiles’ subset.

The final iteration of each model was bootstrapped (nboot =  10,000). The raw and wrangled data, all materials, and model outputs used in this study are openly available on OSF at https://osf.io/akqxn/overview?view_only=9639feeb25614309a2fb8eddcba2fc4. See SM03 R files and SM04 model iterations output in the Supplemental materials for the full analytical report and results. See SM04 model iterations output in the Supplemental materials for the full analytical report and results of the different models tested. This study was not preregistered.

### Results

**Figure 2.**
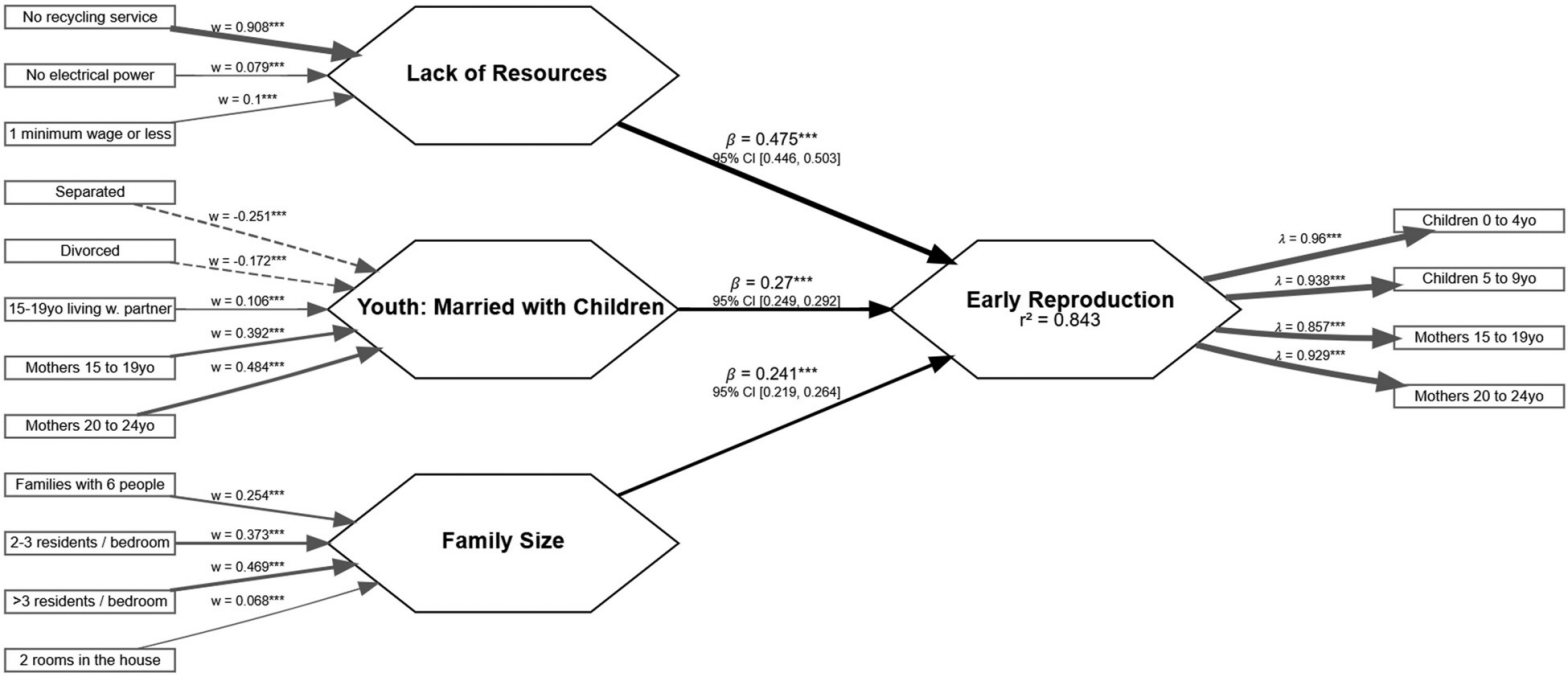
Model predicting early reproduction in Brazil.

*Note.* Figure created in R using seminr package. Hexagons represent latent variables and rectangles represent items from census. Predictors are formative latent variables using census data in 2000 and outcomes are reflective latent variables using census data in 2010.

The selected model used three variables *Youth and married with children*, *Family/house size*, and *Low income and lack of resources* to predict the outcome variable *Early reproduction* (Figure 2). The paths of two variables, *Unemployed or precariously employed* and *Low education*, were negligible (*β* < .1), and the explanatory power of *Youth Married* was also negligible (adj. *R*^2^ < 0.01), so these were dropped from the model. Even though the path from *Low education* to *Early reproduction* was negligible (*β* = .06), it remained statistically significant (*P* < .001), whereas the path from *Unemployed or precariously employed* to *Early reproduction* was not (*P* > .05). Maintaining the two variables in the model to predict *Early reproduction* did not increase the model's explanatory power (increase in adj. *R*^2^ < 0.01). They were, therefore, dropped from the model in accordance with the analytical plan. (For a comparison of the models including and excluding these variables, see Analyses3 and Analyses4 HTML files in sm04_model_iterations_output and their associated output CSV files in the Supplemental materials.)

#### Measurement Model

##### Reflective Measurement Model

*Early reproduction* was defined as a reflective latent variable. In this step, we are assessing if or which variables load into factors to determine what such factors are measuring. Alternatively, a variable can be assigned as a single-item measure if it is an important measure for the research question and it does not load into any factor (see Figure 1). The loadings of the variables in *Early reproduction* are expressed in Figure 2. The indicator reliability values obtained were *Children 0 to 4 years old* = 0.92, *Children 5 to 9 years old* = 0.88, *Mothers 15 to 19 years old* = 0.74, *Mothers 20 to 24 years old* = 0.86. Internal consistency (α = .94, ρ_C_ = .96, ρ_A_ = .95) and reliability (average variance extracted (AVE) = 0.85) were also above the threshold. Heterotrait–monotrait (HTMT) did not pass the recommended threshold (for *Family/house size* HTMT was 0.93, for *Low income and lack of resources* it was 0.90, and for *Youth and married with children* it was 0.96), but Fornell–Larcker criterion did (√AVE = 0.92 and correlations with *Family/house size* = 0.83, *Low income and lack of resources* = 0.89, and *Youth and married with children* = 0.81). Implications of the HTMT and Fornell–Larcker different reports about the variable's discriminant validity are discussed in the Discussion section.

##### Formative Measurement Model

*Youth and married with children*, *Family/house size*, and *Low income and lack of resources* were defined as formative latent variables. The assessment included collinearity (variance inflation factor (VIF) < 5), weights, and loadings for significance and relevance of indicators. Table 2 reports these indices. All indicators were statistically significant (*P* < .01), and the CI did not cross 0.

**Figure 3.**
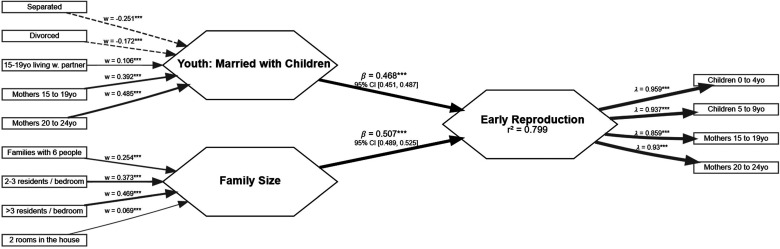
Model predicting early reproduction in Brazil excluding lack of resources.

*Note.* Figure created in R using seminr package. Hexagons represent latent variables and rectangles represent items from census. Predictors are formative latent variables using census data in 2000 and outcomes are reflective latent variables using census data in 2010.

**Table 2.** Assessment of Brazilian Formative Latent Variables.

Latent Variables		VIF	Weights	Loadings
Youth and married with children				
	Separated	1.90	−0.25	−0.51
	Divorced	1.87	−0.17	−0.41
	15–19 years old living w. partner	2.31	0.10	0.73
	Mothers 15 to 19 years old	2.50	0.39	0.81
	Mothers 20 to 24 years old	1.69	0.488	0.84
Family/house size				
	Families with 6 people	1.94	0.25	0.80
	2–3 residents/bedroom	2.80	0.37	0.90
	>3 residents/bedroom	2.80	0.47	0.92
	2 rooms in the house	1.36	0.07	0.43
Low income and lack of resources				
	No recycling service	1.37	0.91	0.99
	No electrical power	1.86	0.08	0.59
	1 minimum wage or less	1.71	0.10	0.54

*Note.* VIF: variance inflation factor (collinearity assessment). Bootstrapped weights and loadings were all significant (*P* < .01) and confidence intervals did not cross zero.

#### Structural Model

The structural model was also assessed for collinearity (VIF < 5), path statistical significance (*P* < .01) and relevance (*β* > 0.1), explanatory power (adj. *R*^2^ ≥ 0.25), and effect size (*f*^2^ ≥ 0.02). Variables in the model met criteria for all three measures, meaning they were not collinear, significant, and relevant predictors of *Early reproduction*. Paths CIs did not cross 0. Table 3 reports these indices. Note that *Low income and lack of resources* was collinear with *Early reproduction* (VIF = 5.14). We tested a model removing this variable (Figure 3) and discussed the implications of these in the discussion section.

**Table 3.** Assessment of Brazilian Structural Model.

Predictors	Early Reproduction		
	VIF	Paths	*f* ^2^
Youth and married with children	2.74	0.27	0.16
Family/house size	3.48	0.24	0.11
Low income and lack of resources	5.14	0.48	0.28
Adj. *R*^2^	0.84		

*Note.* VIF: variance inflation factor (collinearity assessment). Bootstrapped paths were all significant (*P* < .01) and confidence intervals did not cross zero.

Predictive power was assessed with *k*-fold cross-validation model (*k* = 10) with root mean square error out-of-sample between the PLS-SEM model and a naive linear regression model ignoring the latent variables. The performance was similar, but the naive linear regression model performed better than the PLS-SEM (*Children 0 to 4 years old* = 0.638, *Children 5 to 9 years old* = 0.532, *Mothers 15 to 19 years old* = 0.659, *Mothers 20 to 24 years old* = 1.126, and *Children 0 to 4 years old* = 0.666, *Children 5 to 9 years old* = 0.559, *Mothers 15 to 19 years old* = 0.669, and *Mothers 20 to 24 years old* = 1.160, respectively). Mean absolute error out-of-sample statistics were similar, with the linear model performing slightly better than the PLS-SEM model.

#### Model Comparisons

To test whether ethnicity was a predictive factor of early reproduction, we collected data on the percentage of Black, Indigenous, and South Asian people in municipalities. The percentage of Indigenous and South Asian people was mostly absolute zeros in almost half of the municipalities (>2,000 cases), and these measures were removed. The percentage of Black people was then included as a single item both as a predictor and as a mediator in our most restricted model between *Youth and married with children* and *Family/house* and *Early reproduction*. Considering our cut-off criteria, Black ethnicity was neither a relevant predictor (*β* < .01) nor mediator (i.e., direct paths being greater than the multiplication of mediated paths) of *Early reproduction*. *Youth and married with children* path to *Early reproduction* was *β* = .47, whereas the mediated path was *β* = –.09 to Black ethnicity and *β* < .01 to *Early reproduction*, which results in a mediated path lower than −.001*. Family/house size* path to *Early reproduction* was *β* = .51, whereas the mediated path was *β* = –.44 to Black ethnicity and *β *< .01 to *Early reproduction*, which results in a mediated path lower than −.001. The path from Black ethnicity to *Early reproduction* was also not statistically significant.

The second planned comparison was to divide the sample between the quartiles with the highest and lowest percentage of children aged 0–4 and of children aged 5–9. There were minor issues with some indices in some of the subsamples (i.e., *Mothers 15 to 19 years old* loading: λ < 0.7 in the lowest quartiles and *Low income and lack of resources* and HTMT crossing 1 in the highest percentage of children aged 5–9). An optimal model (i.e., passing all test criteria) was not sought for these comparisons because we intended to maximize the similarity between these and the first model. Paths, effect sizes, and predictive values were similar between the different subsamples, but the *R*^2^ was different between the quartiles with highest percentage of children aged 0–4 and 5–9 (adj. *R*^2^ = 0.64 and 0.69, respectively) and the quartiles with the lowest percentage of children in this age period (adj. *R*^2^ = 0.53 and 0.59, respectively).

### Discussion

The first aim of this study was to test if an exploratory analytical approach using LHT-P as the theoretical approach to data selection and interpretation and using the census could successfully predict reproductive patterns in an entire country's population. To do this we tested whether census measures associated with familial socioeconomic stressors, young marriage, and living in a poorly resourced area could predict early reproduction as reported in the census 10 years later. The explanatory power for *Early reproduction* was remarkable (adj. *R*^2^ = 0.84). The model was useful in discriminating between factors that were statistically significant (*P* < .01) and relevant (*β* > .1) predictors and factors that were not. The model was also able to discriminate between outcome factors that had acceptable explanatory power and effect sizes from those that did not. We have found that the factors *Low income and lack of resources*, *Youth and married with children*, and *Family/house size* were predictive of *Early reproduction*, whereas other factors were not predictive (e.g., *Low education*), and other outcomes were not predicted (e.g., *Youth married*)*.* Thus, this approach to studying LHT-P has been supported, and further research could attempt to replicate these results by using a similar analysis (e.g., PLS-SEM, exploratory factor analysis) with data from different years, different data sets, or by using a more confirmatory approach.

The second aim was to identify which of the indicators of harshness and unpredictability would predict faster or earlier reproduction. Both the percentage of children aged 0 to 9 years in the population and the percentage of women 15–24 years old with children in the population of Brazilian municipalities can be substantially predicted by the percentage of (1) families with low resources (i.e., no electrical power or garbage collection service and living with one minimum wage or less); (2) women 15–24 years old with children and teenagers with a spouse, and people divorced or separated; and (3) large families with more than one person per bedroom in the previous census. Regardless of these variables being grouped into factors, these were the most successful predictors of earlier reproduction in Brazilian municipalities.

Notably, these effects were measured across a 10-year gap, with predictors measured in 2000 and outcomes measured in 2010. This time delay was chosen because of the assumed developmental phenomenon between harsh and unpredictable environments and later reproductive timing (Ellis et al., 2003; Simpson et al., 2012; Szepsenwol et al., 2019). Based on PAT, our hypothesis was that children experiencing harsh and unpredictable conditions in the first years of life are likely to adopt a “faster” LHS, experience puberty earlier, and start reproducing earlier. Our findings, in addition to findings from previous research, support the assumption that there is a critical period of development (0–7 years old) in which exposure to harsher and unpredictable environments is likely to cue individuals into reproducing more frequently or earlier. Other findings in the literature also support this assumption by showing that sudden or current change in harsh life conditions does not impact reproductive behavior (Richardson et al., 2020) or is associated with reproductive decline (Nolin & Ziker, 2016).

To further support the hypothesis that the observed association might be a developmental phenomenon and not due to some spatial association or some statistical artifact, we repeated the analyses using the quartiles with the highest and lowest percentages of children aged 0–4 and the quartiles with the highest and lowest percentages of children aged 5–9. The paths from predictors to outcome remained similar across the four subsamples (ranging from 0.35 to 0.48) and comparable to the main model, indicating a stability in the association between these variables. Crucially, the municipalities with the most children had greater explanatory power than those with fewer children, which is consistent with the idea that there is a developmental phenomenon.

Because we are using the census data, our analyses used municipal populations, not just a sample of the population. Therefore, conclusions can and should be drawn regarding nonpredictive variables, especially when there is a strong prediction that these variables will be associated with *Early reproduction*. Two variables were worth noting: *Unemployed or precariously employed* and *Low education*. *Unemployed or precariously employed* was not a relevant nor a statistically significant predictor of *Early reproduction*. The variables composing the factor indexed youth who were not economically active and those who were precariously employed or not employed full time, so we intended it as a factor indicative of parental employment change, which is common in the PAT literature as a measure of unpredictability (Belsky et al., 2012; Ellis et al., 2003; Hartman et al., 2018). In the third iteration of the model, the CI of the variable's weights (i.e., indicator of variable's statistical significance) included 0, suggesting that they were not statistically significant to describe the factor. In addition, the paths from *Unemployed or precariously employed* to both *Married* and *Early reproduction* were below 0.1, suggesting that it was not a relevant predictor to both of our outcome variables. This is surprising given that Brazil faces a social security problem of informality in work. Depending on classification criteria, between 45% and 55% of Brazilian employees were working informally in 2004, and informality was negatively associated with age (Henley et al., 2009). The fact that a higher percentage of young males and females who were not economically active or were precariously employed was not a relevant predictor of teenage or young adults with children is contrary to some previous reports (Belsky et al., 2012; Simpson et al., 2012), but consistent with another (Hartman et al., 2018).

*Low education*, which could be an indicator of lack of access to resources, was a statistically significant but not a relevant predictor of* Early reproduction*. In Brazil, education attainment has also been negatively and significantly associated with informal employment in the 1990s and 2000s (Henley et al., 2009). Using England and Wales Census data, Copping and colleagues (Copping et al., 2013; Copping & Campbell, 2015) have found an association between education and family instability and LHSs. In addition, women's access to education has been associated with global fertility decline (Roser, 2014). One possible explanation for these variables not being a relevant or statistically significant predictors of *Early reproduction* may be the presence of other variables that better explain the phenomenon (e.g., *Low income and lack of resources*). Lower education attainment and employment precariousness are expected to be associated with lower pay and reduced access to resources. The absence of such variables in previous research may explain the difference between them being significant predictors of LHSs.

#### Caveats

Proper interpretation of these findings should be mindful that this research has used aggregated municipal data to assess hypotheses regarding individuals' developmental processes. Finding patterns present in entire populations is a valuable contribution to PAT. However, aggregate municipal data is far from being ideal to assess these hypotheses, and these limitations are discussed in the General Discussion section.

Ten years is not an ideal time frame to test this developmental prediction. Children 0–7 years old experiencing harsh or unpredictable environments would be 10–17 years old by the time we assessed early reproduction. Regarding the outcomes, the parents of children 5–9 years old could not have been in the critical period of development of 0–7 years a decade earlier, especially for children aged 7–9 years. To offer some insight into this issue, we ran a post hoc model removing the variable *children 5–9 years old* from *Early reproduction*. The model performed similarly to the selected model. Differences were smaller than 0.1 in the exploratory power (*R*^2^ = 0.79), paths from predictors to outcome (*β* > .2) and factor loadings (λ > 0.8). Nevertheless, interpreting these findings within PAT assumptions would also assume that these indicators are somewhat stable over time. Harsher and more unpredictable municipalities in Brazil in 2000 would also have been harsher and more unpredictable a few years prior.

Some parameters in the model of the study required theory-informed interpretation. One of our discriminant validity measures (HTMT) was not attained for the three predictors. We decided to retain it because the other measure (Fornell–Larcker) passed the criteria and because the issue with a high HTMT is the risk of it being above 1 in the population (Hair et al., 2022). Bootstrapped measures did not reach 1; therefore, it is very unlikely that the HTMT is 1 in the population, which suggests that the variables attained discriminant validity.

Second, *Low income and lack of resources* was substantially collinear with *Early reproduction* (VIF = 5.14), which would favor removing the variable. We decided to retain the variable in the model because the explanatory power between the model including *Low income and lack of resources* and the model excluding it were similar (*R*^2^ = 0.84 and 0.80, respectively). In addition, there is an association between harshness, typically measured as socioeconomic status (Chang et al., 2019; Doom et al., 2016), and “faster” LHS (Ellis et al., 2009). Third, considering subject matter knowledge should be applied to model selection (Harrell, 2015), especially in exploratory research. We argue that the variables here are conceptually different. For example, not having electrical power or garbage collection services and living with low income are conceptually different from being a young mother or living in a large family (the other predictors) and also different from our *Early reproduction* measures. It also helps in the interpretation that the observed collinearity—or a high degree of association—did not occur between our measures of *Youth and married with children* variable and the measures in *Early reproduction*, which are conceptually similar to each other, and the path between them was not closest to 1. A Pearson correlation analysis also did not show a correlation close to 1 between women 15–24 years old with children in 2000 with the same variable in 2010 (*r* = .58, *P* < .001). The percentage of women 15–19 years old in 2010, for example, was more highly correlated with the predictors *No recycling service*, the percentage of children 0–4 years old, and the percentage of children 5–9 years old (*r* > .68, *P* < .001), but the latter two were not included in the model. If the data was only observing a geographical correlation across time, one would expect collinearity and a very high correlation among variables that are conceptually the same variable but that were measured at two time points. This again also suggests that we are not observing a mere geographical correlation. It is likely that certain municipalities with a higher percentage of children aged 5–9 years old living in harsh conditions and with larger families then see these children grow up and become young parents. Future research may repeat this study with census data in different years or make some changes in the variables composing the factors to assess whether this collinearity persists.

For the comparison of ethnicity, however, we removed the variable *Low income and lack of resources* and tested whether Black ethnicity would be a relevant predictor or mediator of *Early reproduction*. As stated above, we expected nearly all variables to be statistically significant, even in adopting *P* < .01; therefore we established a *β* > .1 as a threshold for considering a variable relevant. Surprisingly, though, even though Brazil has both historical and institutional anti-Black racism (Couto & Brenck, 2024; Pimentel, 2022), the percentage of Black people was not a relevant predictor nor a mediator of *Early reproduction*, which was contrary to our fourth hypothesis. We believe that this may be due to the percentage of people in Brazilian municipalities identifying as Black (mean = 5.8) being insufficient to impact the geography measure. In fact, most Brazilians self-identify as “Pardos” (roughly translated to Brown). Adding “Pardos” and Blacks as a variable or racial visible minority could have increased the ratio but could also reduce variability across municipalities and introduce noise, so we decided not to add them in this study. Future studies could explore this further by using the percentage of specific ethnicities as a predictor variable.

Assessing the predictive value of the model is a recommended step of PLS-SEM (Hair et al., 2019, 2022). In this step, the prediction of each of the directly observed variables (i.e., the four variables indicating *Early reproduction* in this model) of our PLS-SEM model is compared to a naive linear regression model (i.e., not converging variables into factors). The linear regression model performed better than the PLS-SEM model in every model we have tested. We believe this is due to the fact that the factors included in our analyses derived from measures that are not sufficiently correlated to capture our hypothesized constructs. The variables measuring any of our factors were not originally intended to measure *Early reproduction*, *Low income and lack of resources*, *Family/house size*, nor *Youth and married with children*. In this study, they were grouped into factors because of a hypothesized association derived from LHT-P literature and statistically derived from the PLS-SEM measurement model measures to justify such groupings. In this case, they are illustrative of the common harshness of unpredictability conditions that can predict a faster LHS (Ellis et al., 2009). Should a linear model be a better predictor of such a process, it is of little harm to our hypotheses.

In addition, the predictive value assessment is meant to be indicative of the capacity of the model to predict out-of-sample outcomes (Hair et al., 2022; Shmueli et al., 2016). However, because this study is using census data, there is no alternative sample where this model can be replicated. We are not estimating what would be the association between predictors and outcomes in a population; we are describing what they are. This is another reason why we did not rely much on statistical significance when choosing the optimal model. Moreover, previous research has not reported the predictive value of PLS-SEM models (Kazár, 2014) or has tested the predictive value of PLS-SEM models with different approaches (Shmueli et al., 2016; see Riou et al., 2016 for an example).

Municipalities likely have higher correlations between variables when those municipalities are nearer geographically. It is possible that the predictive value has been affected by this spatial relationship. In addition, characteristics specific to a region, community, or the differences between urban and rural communities could confound the interaction between the variables studied, increasing error in the model prediction. Future studies using spatial cross-validation (Pohjankukka et al., 2017) or stratified cross-validation (Diamantidis et al., 2000) approaches could address this issue.

A final limitation of this study is its exploratory nature (Szollosi & Donkin, 2021). We used PAT to inform variables’ selection and, to the extent possible, the time frame of a phenomenon. Therefore, this study is descriptive and not ideal for hypothesis testing. Future studies could attempt to use more confirmatory approaches, CB-SEM for example. When supported by theoretical background, CB-SEM allows for hypothesized causal inferences (Kline, 2016; Maruyama, 1997). Indeed, there has been a claim for more formal and more refined models in LHT in psychology (Nettle & Frankenhuis, 2020; Stearns & Rodrigues, 2020). The second study in this article attempted to use CB-SEM with a different data set (ACS). Future studies could test whether this pattern is representative of a more longitudinal/developmental phenomenon or a mere geographical correlation by using same-year (i.e., predictors and outcomes extracted from the same census year) or reverse (i.e., predictors extracted from the most recent census and outcomes extracted from the past) models. These comparisons, however, are beyond the scope of this article.

## Frequent Reproduction in the United States

In this study, we used a confirmatory method of analysis to determine if variables similar to the significant predictors of reproduction patterns in Brazilian municipalities would be significant predictors of reproductive indicators in a new data set. We chose a different country for this test (United States) and a different year range. We hypothesized that most of the variables would be significant predictors of reproduction.

### Methods

#### Data Selection and Transformation

We utilized the variables that were relevant and significant predictors or outcomes in the first study (Figure 2) to explore potential variables for this study. Publicly available data was collected from the ACS: 5-Year Estimates aggregate tables (U.S. Census Bureau, 2020) using the Census Bureau of Statistics API. Predictors were extracted from the ACS 5-year aggregate table in 2009 (i.e., aggregate estimates from 2005 to 2009) and outcomes in 2023 (i.e., aggregate estimates from 2019 to 2023). This represented a time difference of around 14 years between predictors and outcomes. The ACS produces its estimates by collecting data from around 3.5 million addresses in the United States and Puerto Rico every year. These are reported as estimates of geographies with at least 65,000 people (e.g., census tracts or counties). The 5-year aggregate combines data from 5 consecutive years to produce estimates of geographies with fewer than 65,000 people (U.S. Census Bureau, 2020).

US counties were decided to be the geographic level of data collection. Counties or equivalents are the primary divisions of American states, and they usually include multiple cities or towns (U.S. Census Bureau, 2022). The choice of counties instead of smaller geographies is intended to reduce the likelihood of a high percentage of the population moving during the 14-year time span under which data was collected.

Data from ACS 2009 included 3,221 counties, and data from ACS 2023 included 3,222 counties of the US mainland, Alaska, Hawaii, and Puerto Rico. Table 4 reports the 25 variables that were collected for this study and how they compare to the variables that were used in the model in the first study. Similar to the study utilizing the Brazilian Census, variables were square-root and log-transformed, and the boxplots of these variables were used to choose the transformation (or non-transformed variable) that most closely resembled a normal distribution. Outlier (*z*-score > |3|) and NA removal (≥5%) were also used in the same way as in the first study.

**Table 4.** Variables in the Model Using Brazilian Census Compared to Variables Using US American Community Survey.

Variables in Brazilian Census Geographic Level: Municipalities	Variables Found in the American Community Survey (ACS) Geographic Level: Counties	Classification Equivalent (E) Similar (S) Not Found (N)	ACS Code
Predictors: 2000	Predictors: 2005–2009		
(lacking) Existence of services and durable goods—recycling or waste collection	Lacking complete plumbing facilities^a^; Plumbing facilities for occupied Housing Units	S	B25048_003E
(lacking) Existence of services and durable goods—electric lights	Lacking complete kitchen facilities^a^; Kitchen facilities for occupied housing units	S	B25052_003E
People with income of 1 minimum wage or less	Income in the past 12 months below poverty level^b^	S	B17001_002E
Separated—judicially separated	Separated^a,c^: Male; Now married; Married, spouse absent; Separated+ Female; Now married; Married, spouse absent; Separated	E	B12001_007E B12001_016E
Divorced	Divorced^b,c^: Male; Divorced+ Female; Divorced	E	B12001_010E B12001_019E
Age group—15 to 19 years of age—Living with a spouse or partner	Women who did not have a birth in the past 12 months; Now married (including separated and spouse absent); 15 to 19 years old^b^	S	B13002_013E
Age group - 15 to 19 years of age—with children	Women who had a birth in the past 12 months; Now married (including separated and spouse absent); 15 to 19 years old+ Women who had a birth in the past 12 months; Unmarried (never married, widowed, and divorced); 15 to 19 years old^b,c^	S	B13002_004E B13002_008E
Age group—20 to 24 years of age—with children	Women who had a birth in the past 12 months^a^	S	B13002_002E
Number of family members—6 people		N	
Residents density per dormitory—more than 2.0 to 3.0 residents	Complete plumbing facilities; 1.01 or more occupants per room^b^	S	B25050_007E
Residents density per dormitory—more than 3.0 residents		N	
Number of rooms—2 rooms	Median number of rooms	S	B25018_001E
Black ethnicity	Sex by age (Black or African American alone)^a^	E	B01001B_001E
Indigenous ethnicity	Sex by age (Hispanic or Latino)^a^	N	B01001I_001E
Age group—0 to 4 years of age	Age group—0 to 4 years of age^c^: Male; Under 5 years+ Female; Under 5 years	S	B01001_003E B01001_027E
Age group—5 to 9 years of age	Age group—5 to 9 years of age^c^: Male; 5 to 9 years+ Female; 5 to 9 years	S	B01001_004E B01001_028E
Outcomes: 2010	Outcomes: 2018–2023		
Age group—0 to 4 years of age	Age group—0 to 4 years of age^c^: Male; Under 5 years+ Female; Under 5 years	S	B01001_003E B01001_027E
Age group—5 to 9 years of age	Age group—5 to 9 years of age^c^: Male; 5 to 9 years+ Female; 5 to 9 years	S	B01001_004E B01001_028E
Women 15 to 19 years of age with children	Women who had a birth in the past 12 months; Now married (including separated and spouse absent); 15 to 19 years old^b^	S	B13002_004E
Women 20 to 24 years of age with children	Women who had a birth in the past 12 months	S	B13002_002E

*Note.* ACS code denotes the code used by the US Census Bureau to identify the variable.

a Log-transformed variables.

b Square root-transformed variables.

c Manually calculated the sum of the variables to come up with a single variable representative of both groups.

Merging the data frames (2009 and 2023) resulted in 3,209 counties. The reduction from the original data frames (e.g., 3,221 in 2009) is usually due to the division or redefinition of a county, which would result in a different code for this new geographical division. Outlier removal resulted in a data frame of 2,763 counties, which represents a reduction of around 15% of the data frames that were collected or merged. No variable had an NA count higher than 5%; therefore, no variables were removed due to this process. The final data frame consisted of 19 predictor and 6 outcome variables and 2,763 cases. All scripts and a full report of data curation and transformation are available in the Supplemental materials.

### Results

CB-SEM analysis was planned for this data. CB-SEM is an alternative to PLS-SEM that encompasses a more confirmatory approach (Hair et al., 2022; Kline, 2016). The measurement model is tested in confirmatory factor analysis, and in a subsequent step the structural model is tested with the paths between factors.

The confirmatory factor analysis step, however, did not reach a solution in different attempts to load the variables into factors. These attempts were composed of different variable organizations (as long as there was theoretical support in LHT-P) and of using variables that ACS reports separated by sex, both separately and added. The solutions consistently presented one variable with high loading (e.g., λ > 1) while the others had low loadings (e.g., λ < 0.3). See the Supplemental materials for a full report of these steps. Since we are using simple models (i.e., without mediation or multiple latent outcome variables), removing the factors rendered our CB-SEM analysis as only a series of multivariate linear regressions. Therefore, the analytical approach of US data was switched to multivariate linear regressions.

Different multivariate linear regressions used the outcome and predictor variables described in Table 2. Visual inspection of diagnostic plots indicated that the residuals were approximately normally distributed, with some deviations in the tails and with slight heteroskedasticity. Given that this is demographic data with non-normal distribution and the arguably large sample size, we utilized heteroskedasticity-consistent degrees of freedom correction (HC1) in order to inflate residuals and make the analysis more reliable (Long & Ervin, 2000; MacKinnon & White, 1985). We assessed collinearity with VIF > 3, but no predictor was removed because none was above this criterion. Nonsignificant variables were removed from the model until only statistically significant variables were left, a process similar to stepwise linear regression analysis (Harrell, 2015).

Tables 4 to 7 report the models for each of the outcome variables, and Table 8 reports the explanatory power of these four models. The models predicted a substantial proportion of variance of *Age Group—0 to 4 Years of Age* (*R*^2^ = 0.49, *F*(11, 2,733) = 240.91, *P* < .001, adj. *R*^2^ = 0.49) and of *Age Group—5 to 9 Years of Age* (*R*^2^ = 0.39, *F*(6, 2,738) = 291.82, *P* < .001, adj. *R*^2^ = 0.39) and a weak or negligible proportion of variance of *Women 15 to 19 Years of Age Who Had Birth* (*R*^2^ = 0.07, *F*(5, 2,739) = 43.71, *P* < .001, adj. *R*^2^ = 0.07) and of *Women Who Had Birth in the Past 12 Months* (*R*^2^ = 0.10, *F*(3, 2,741) = 98.28, *P* < .001, adj. *R*^2^ = 0.10). Frequency tables showing the mean of each outcome variable for each tertile of the predictor variables were also compiled to provide a better visualization of the models (Tables 9 to 13). In these frequency tables, the means are expressed without transformation (i.e., log or square root).

**Table 5.** Variables Predicting Percent of 0- to 4-Years Olds in US Counties.

Effect	*β*	SE	*t*-Value	*P*-Value	Std. *β*
Intercept	.38	0.48	0.80	.426	
Lacking complete plumbing facilities	−.34	0.09	−3.81	≤.001***	−.08
Lacking complete kitchen facilities	.38	0.08	4.57	≤.001***	.10
Income in the past 12 months below poverty level	.16	0.03	5.21	≤.001***	.12
Divorced	−.35	0.06	−5.88	≤.001***	−.11
Women who had a birth in the past 12 months	.31	0.11	2.75	.006**	.06
1.01 or more occupants per room	.19	0.06	3.09	.002**	.08
Age group—0 to 4 years of age	.43	0.03	14.11	≤.001***	.51
Age group—5 to 9 years of age	.13	0.03	5.20	≤.001***	.14
Black or African American alone	−.08	0.01	−6.32	≤.001***	−.10
Hispanic or Latino	−.10	0.02	−4.21	≤.001***	−.09
Median number of rooms	.32	0.06	5.11	≤.001***	.13

*Note.* *P ≤ .05; **P ≤ .01; ***P ≤ .001.

**Table 6.** Variables Predicting Percent of 5- to 9-Years Olds in US Counties.

Effect	β	SE	*t*-Value	*P*-Value	Std. *β*
Intercept	.59	0.42	1.43	.154	
Divorced	−.17	0.07	−2.63	.009**	−.05
Women who had a birth in the past 12 months	.27	0.13	2.15	.031*	.05
Age group—0 to 4 years of age	.43	0.03	14.79	≤.001***	.45
Age group—5 to 9 years of age	.19	0.03	6.27	≤.001***	.18
Black or African American alone	−.13	0.02	−8.41	≤.001***	−.14
Median number of rooms	.35	0.05	6.94	≤.001***	.12

*Note.* *P ≤ .05; **P ≤ .01; ***P ≤ .001.

**Table 7.** Variables Predicting Percent of 15- to 19 Years of Age Who Had Given Birth.

Effect	*β*	SE	*t*-Value	*P*-Value	Std. *β*
Intercept	−.17	0.03	−5.79	<.001***	
Lacking complete kitchen facilities	.03	0.01	2.97	.003*	.06
Divorced	.03	0.01	3.95	<.001***	.08
Women 15 to 19 years of age who had birth	.05	0.02	3.25	.001***	.07
Age group—0 to 4 years of age	.02	0.00	8.91	<.001***	.18
Black or African American alone	.01	0.00	4.33	<.001***	.09

*Note.* *P ≤ .05; **P ≤ .01; ***P ≤ .001.

**Table 8.** Multivariate Linear Regression Model of Women Who Had Given Birth in the Past 12 Months.

Effect	*β*	SE	*t*-Value	*P*-Value	Std. *β*
Intercept	.13	0.06	2.25	.024*	
1.01 or more occupants per room	.03	0.01	2.42	.016*	.06
Age group—0 to 4 years of age	.04	0.00	9.76	<.001***	.18
Median number of rooms	.06	0.01	6.38	<.001***	.09

*Note.* *P ≤ .05; **P ≤ .01; ***P ≤ .001.

**Table 9.** Explanatory Power of the Four Models.

Outcome Variable	*R* ^2^	Adj. *R*^2^	*F* Statistic	*P*-Value
Age group—0 to 4 years of age	0.49	0.49	*F*(11, 2,733) = 240.91	<.001
Age group—5 to 9 years of age	0.39	0.39	*F*(6, 2,738) = 291.82	<.001
Women 15 to 19 years of age who had birth	0.07	0.07	*F*(5, 2,739) = 43.71	<.001
Women who had birth in the past 12 months	0.1	0.1	*F*(3, 2,741)	<.001

*Note.* All regression models were statistically significant at *P* < .001.

**Table 10.** Percent of Age Group—0 to 4 Years of Age by Low, Medium, and High Tertile of Predictors.

Outcome Variable	Low Tertile	Middle Tertile	High Tertile	*P*-Value
Lacking complete plumbing facilities	5.48	5.54	5.56	***
Lacking complete kitchen facilities	5.39	5.51	5.52	***
Income in the past 12 months below poverty level	5.40	5.52	5.50	***
Separated	5.54	5.44	5.43	
Divorced	5.78	5.46	5.17	***
Now married—15 to 19 years old	5.38	5.45	5.60	
Women 15 to 19 years of age who had birth	5.23	5.49	5.70	
Women who had a birth in the past 12 months	4.98	5.42	6.03	**
1.01 or more occupants per room	5.13	5.46	6.00	**
Age group—0 to 4 years of age	4.76	5.41	6.25	***
Age group—5 to 9 years of age	4.88	5.49	6.06	***
Black or African American alone	5.56	5.33	5.52	***
Hispanic or Latino	5.36	5.47	5.58	***
Median number of rooms	5.30	5.55	5.61	***

*Note.* *P ≤ .05; **P ≤ .01; ***P ≤ .001.

**Table 11.** Percent of Age Group—5 to 9 Years of Age by Low, Medium, and High Tertile of Predictors.

Outcome Variable	Low Tertile	Middle Tertile	High Tertile	*P*-Value
Lacking complete plumbing facilities	6.01	5.96	6.02	
Lacking complete kitchen facilities	5.93	5.91	6.04	
Income in the past 12 months below poverty level	6.00	5.96	5.92	
Separated	6.08	5.98	5.81	
Divorced	6.27	5.93	5.67	**
Now married—15 to 19 years old	5.90	5.91	6.07	
Women 15 to 19 years of age who had birth	5.77	5.93	6.18	
Women who had a birth in the past 12 months	5.44	5.93	6.51	*
1.01 or more occupants per room	5.66	5.91	6.42	
Age group—0 to 4 years of age	5.24	5.92	6.72	***
Age group—5 to 9 years of age	5.30	5.98	6.61	***
Black or African American alone	6.14	5.84	5.90	***
Hispanic or Latino	5.80	5.93	6.15	
Median number of rooms	5.76	5.97	6.19	***

*Note.* *P ≤ .05; **P ≤ .01; ***P ≤ .001.

**Table 12.** Percent of Women 15 to 19 Years of Age Who Had Birth by Low, Medium, and High Tertile of Predictors.

Outcome Variable	Low Tertile	Middle Tertile	High Tertile	*P*-Value
Lacking complete plumbing facilities	0.039	0.035	0.042	
Lacking complete kitchen facilities	0.035	0.037	0.047	*
Income in the past 12 months below poverty level	0.026	0.034	0.057	
Separated	0.030	0.037	0.051	
Divorced	0.039	0.037	0.041	***
Now married—15 to 19 years old	0.040	0.033	0.044	
Women 15 to 19 years of age who had birth	0.034	0.032	0.051	***
Women who had a birth in the past 12 months	0.035	0.036	0.047	
1.01 or more occupants per room	0.027	0.037	0.052	
Age group—0 to 4 years of age	0.032	0.036	0.050	***
Age group—5 to 9 years of age	0.031	0.037	0.050	
Black or African American alone	0.033	0.036	0.048	***
Hispanic or Latino	0.035	0.037	0.046	
Median number of rooms	0.049	0.039	0.027	

*Note.* *P ≤ .05; **P ≤ .01; ***P ≤ .001.

**Table 13.** Percent of Women Who Had Birth in the Past 12 Months by Low, Medium, and High Tertile of Predictors.

Outcome Variable	Low Tertile	Middle Tertile	High Tertile	*P*-Value
Lacking complete plumbing facilities	1.19	1.20	1.17	
Lacking complete kitchen facilities	1.18	1.18	1.17	
Income in the past 12 months below poverty level	1.19	1.18	1.16	
Separated	1.21	1.16	1.16	
Divorced	1.25	1.16	1.12	
Now married—15 to 19 years old	1.17	1.17	1.19	
Women 15 to 19 years of age who had birth	1.16	1.16	1.21	
Women who had a birth in the past 12 months	1.08	1.18	1.28	
1.01 or more occupants per room	1.10	1.19	1.28	*
Age group—0 to 4 years of age	1.06	1.17	1.31	***
Age group—5 to 9 years of age	1.05	1.20	1.28	
Black or African American alone	1.20	1.14	1.19	
Hispanic or Latino	1.15	1.18	1.20	
Median number of rooms	1.12	1.19	1.24	***

*Note.* *P ≤ .05; **P ≤ .01; ***P ≤ .001.

### Discussion

This study aimed to assess whether an analysis with US data would have similar findings to the Brazilian Census analysis, but this time with a confirmatory analysis. There were a few similarities. Linear regressions fitted were capable of explaining a substantial proportion of the variance of the percentage of the population that were children aged 0–4 and 5–9 years in US counties using variables that were also predictive in Brazil and that are akin to variables usually understood to be measures of harshness and unpredictability in PAT (Belsky et al., 2012; Ellis et al., 2003; Hartman et al., 2018; Xu et al., 2018). A higher percentage of children in a given area can be understood as more frequent reproduction. We assume that this higher percentage of children is indicative of one of two things: (1) a higher percentage of women are having children in these counties; or (2) a subset of women in these counties are having multiple children.

Even though the outcome variables of young motherhood (*Women 15 to 19 Years of Age Who Had Birth*) and recent reproduction (*Women Who Had Birth in the Past 12 Months*) had significant predictors, the explanatory power of these variables was weak or negligible. Therefore, we will not consider that the model performed well in predicting these variables and will not detail them in the discussion.

Some considerations can help understand this poor performance. First, there is a global trend toward the decline and delay in fertility (Roser, 2014; Volk, 2023). Access to education and health care, especially among women, are understood to be the lead causes of this decline and delay. Observing the frequency tables, we can see that the non-transformed percentage of *Women 15 to 19 Years of Age Who Had Birth* is always smaller than 0.1%, and of *Women Who Had Birth in the Past 12 Months* is never above 2%, and this global trend could explain the small percentage found in our data.

Second, the notably small percentages, particularly in the first case, mean that outlier cases potentially resulting from confounding variables would exert a disproportionately large influence on the variance of these variables. This noise would likely have a reduced impact if these outcome variables encompassed a greater proportion of the population. Lastly, the considerable right-skewness observed (i.e., values clustering near zero) increases the likelihood of the model being impacted by the violation of distributional assumptions required for linear regression analyses. Future studies could repeat this analysis with a subset of the population in which there are higher percentages of young or recent parents or make use of a more robust analysis.

The model predicting the percentage of *Age Group—0 to 4 Years of Age* in 2023 contained 11 significant predictors, and the one predicting *Age Group—5 to 9 Years of Age* contained six predictors. Out of these, the outcome variable (i.e., the percentage of children in these age ranges) was also a predictor. This brings up the discussion about whether these variables represent merely a geographical correlation across time. However, predictors and outcomes are separated by roughly 14 years; therefore, one can also argue that the process is that US counties with a higher percentage of children (0–9 years old) in 2009 had these children become of reproductive age 14 years later, and a subset of these people had children in 2023, maintaining the higher percentage of children in comparison with other US counties. Interestingly, the percentage of children aged 0–4 years had the highest *t*-values and standardized *β* for both of the outcomes: children aged 0–4 years and children aged 5–9 years. If we were observing merely geographical correlation over time, one would expect the same variables to have the highest associations, which did not happen for children aged 5–9 years.

Fewer resources in US counties in 2009—indicated by not having a complete kitchen, having income below poverty level, and having more than one occupant per room in the household—were positively associated with *Age Group—0 to 4 Years of Age* in 2023. This is in line with the literature that posits that harsher environments will favor having children more frequently (Belsky et al., 2012; Ellis et al., 2009; Stearns, 1992). Contrary to this literature, not having complete plumbing or the median number of rooms in the household was in the opposite direction, negative for the former and positive for the latter. It may be the case that not having complete plumbing in the household is characteristic of a particular setting (i.e., rural areas), in which an association between that and harsher life conditions would not exist or not be sufficiently measurable. The interpretation of the association between household density (occupants per room) and young children can also be ambiguous, as it could be partially due to multiple generations cohabiting. Some young children may be the grandchildren of the primary occupants. For instance, households with more children may also be households with more rooms, but the increase may not be 1 to 1 (i.e., children sharing bedrooms). The median number of rooms was a statistically significant predictor of the percentage of children aged 5–9 years, but having one or more occupants per room was not.

While the percentage of women who gave birth in the past 12 months was positively associated with the percentage of children both aged 0–4 and aged 5–9 years, the percentage of divorced people was a negative and statistically significant predictor of the percentage of children in these age groups. The former may support the claim that the phenomenon being observed is of more frequent reproduction in harsher environment, while the latter is contrary to what has been observed in the literature. One of the explanations of LHT-P is that experiencing parental transitions during childhood would favor faster and more frequent reproductive strategies because it would alter the perception that the parental figure (usually the father) would remain in the relationship and invest in children (Belsky et al., 2012; Volk, 2023). Our data shows that having fewer divorced people in 2009 predicts a higher percentage of children aged 0–9 years.

Differently from the study using the Brazilian Census, a high percentage of Blacks in 2009 was a statistically significant predictor of the percentage of children aged 0–9 years in 2023, and the percentage of Hispanic people was a significant predictor of the percentage of children aged 0–4 years. These associations were all negative, meaning that once you remove the effect of socioeconomic predictors, the ethnicity itself is actually a predictor in the contrary direction of the common perception that these ethnicities have larger families (Pew Research Center, 2015).

This study suffers from similar limitations as the study with Brazil data. Considering that we are describing an observation made with aggregate values of large geographical areas (US counties), one should be very careful when considering how our results apply to individual behaviors. There is the possibility that the associations found at the county level would not be found at the individual level, that confounding variables are present, and that, if observed or measured, they would result in a different interpretation of the phenomenon at the individual level.

Data in both studies were transformed; therefore, *β* values cannot be directly interpreted as a multiplier to convert change in the predictor variable to change in the outcome variable. Future studies could use subsamples that would not require such transformations and develop models that would allow for such interpretation. Even after transformation, data was not normal. PLS-SEM—used in the analysis of Brazil data—is considered robust against non-normality. The data used here, however, was severely skewed, so we opted for the transformation.

Linear regressions—used in the analysis of US data—assume normality of residuals. Transforming the data improved the non-normality and heteroskedasticity, and we also used HC1 correction to obtain robust standard errors. Finally, our sample consisted of more than 2,700 cases, and *P*-values are sensitive to sample size (Gelman & Weakliem, 2009; Lakens, 2022). The statistical significance level reported for most variables were below .001 but they should still be interpreted with caution as they could be merely reflecting an overpowered analysis. Future studies should seek to confirm the results in this study and refine model predictions.

## General Discussion

Our first study determined if predictions derived from LHT-P could account for measures of reproductive behaviors contained in theBrazilian Census data. Studies with a similar approach have been conducted before in the United Kingdom (Copping et al., 2013; Copping & Campbell, 2015), but to our knowledge, this study is novel in a few characteristics. First, the analysis of the Brazilian Census was the first study to utilize population data from a developing country to assess PAT hypotheses. Second, our analyses used a longitudinal approach—using census data from one year to predict data collected a decade or more later—to assess the developmental prediction proposed by PAT (Ellis et al., 2003; Simpson et al., 2012; Webster et al., 2014). Third, this project is the first to compare the results obtained from two countries with very large populations (more than 200 million people). Fourth, the study is also the first to assess whether ethnicity—used as a proxy for harsh and unpredictable circumstances along with other measures common in PAT literature—would be predictive of reproduction.

These two studies were designed to answer several research questions. The first was whether an exploratory approach to a structural equation modeling analysis would converge and perform well using secondary data. This question is interesting because secondary data tend to be suboptimal for variable selection (Andersen et al., 2011; Johnston, 2017; Jones, 2010) and are unlikely to achieve model fit (Hair et al., 2022) because data collection was not designed for that purpose. This analysis is even more challenging because we used variables measured in one year to predict outcomes a decade later.

PLS-SEM analyses of the Brazilian data worked reasonably well. Some variables converged into satisfactory factors (e.g., *Low income and lack of resources*) while others did not (e.g., *Early reproduction* was initially tested with variables of married youth) and some were good predictors (e.g., *Low income and lack of resources*) whereas others were not (*Unemployed or precariously employed*). The structural assessment was also satisfactory, and the explanatory power of *Early reproduction* was substantial to a level that is rarely found in the social sciences. The cross-validation predictive power of the PLS-SEM model was similar to what would be achieved by linear models.

In the study with US data, however, the variables did not converge into factors in the CB-SEM analysis. As discussed above, this nonconvergence might be caused by the nature of the data—secondary data, not intended to be grouped as factors in its conception—or by the nature of the analysis—confirmatory and assuming reflective factors. We believe that PLS-SEM is suitable for analyzing this type of data and encourage future studies to use it to analyze data from different countries.

The second aim of the study was to assess which predictors, among the ones that were akin to those frequently used in LHT-P, would predict variables that index early reproduction. In contrast to studies that identified unpredictability (Belsky et al., 2012; McLaughlin et al., 2021; Simpson et al., 2012), especially parental transitions (Hartman et al., 2018), as the strongest predictor of faster LHSs, our study found that *Low income and lack of resources* was a better predictor of *Early reproduction* than measures of marital status (understood here as a proxy for parental transitions) and measures of unemployment or precariously employment (understood here as a proxy for parental availability).

Most studies in the field have been conducted in developed countries. Such populations may have both lower levels of harshness and less variance in the measures of harshness frequently used in LHT-P (i.e., socioeconomic status). The variance in harshness in our data set may explain why harshness was the best predictor of early reproduction in our study but not in studies relying on data from developed countries.

The variance of the measures of unpredictability used in this study could be obscured by other variables. For example, the same variance or even the same regions in which there is a high level of unemployment (*Unemployed or precariously employed*, intended here to measure parental availability) could covary with *Low income and lack of resources*, which would obscure the predictive power of one of the variables. The collinearity assessment did not show that measures were collinear, which is an outcome that we would expect if they were strongly correlated. However, this assessment only measures collinearity among factors, not among manifest variables. It is possible that a different arrangement of the manifest variables would reveal such collinearity.

The third aim was to assess whether an analysis of survey data from a developed country would yield similar findings to the ones obtained from an analysis of census data from a developing country. Results from Brazil mainly indicated that areas that were associated with harsher conditions reported—10 years later—as having more and younger children. In addition, the percentage of mothers aged 15–24 years and of people 15–19 years old living with a partner were also predictive of a higher percentage of children and of younger mothers. We argue that this is a developmental phenomenon: it is likely that many children living in those municipalities in 2000 grew up to become young mothers in 2010. The results from the United States were comparable. For models predicting the percentage of children aged 0–4 and children 5–9 year indicators of harshness (e.g., as lacking a complete kitchen, low income, more rooms per dwelling, more than one occupant per room), and a higher percentage of women who had births in past 12 months and the percentage of children aged 0–9 years old were predictive of the percentage of children aged 0–4 years old. In this study, predictors and outcomes were separated by roughly 14 years. This, once again, may support the developmental hypothesis that many children living in counties with higher levels of harshness in 2009 grew up to become parents in 2021.

In both studies, the percentage of divorced people was a negative predictor of the percentage of children. This result was surprising because “parental transitions” is a common measure associated with a faster LHS in LHT-P (Belsky et al., 2012; Ellis et al., 2009; Hartman et al., 2018). Parental transitions have been assumed to be a signal to children raised in these environments that relationships do not last, so one should expect lower partner investment in raising children, but whether this is really a cue has been questioned recently (Volk, 2023). This result is contradictory to PAT assumptions. It can be interpreted, though, in light of the recent shifts of marriage and divorce statistics (Herre et al., 2020; Kennedy & Ruggles, 2014). Marriage has been in a slight decline and there is an increase in conjugal relationships and parenting without marriage, which in turn lowers divorce rates. Marriage and divorce are also happening at later age. These could decouple the measure of divorce rates from the parental transition it was intended to measure. In addition, divorce in modern environments and divorce in our environment of evolutionary adaptedness can result in considerably different outcomes for children (Ellis et al., 2009; Volk 2023). In the environment of evolutionary adaptedness, the absence of a parent early in life is much more likely to decrease chances of survival, which is the original measure of harshness in PAT research. All of these can impact the predictive power of divorce rates.

Another comparison between the two countries that are of interest is the direction of the prediction. Results from Brazilian Census indicated that lack of access to recycling service and to electrical power was associated with a higher percentage of children and of early parenting. Lacking plumbing in the United States, however, was a negative predictor of the percentage of children 14 years later. This may be due to its reduced prevalence or more closely related to geographical conditions (e.g., rural environments) instead of an indication of a harsher environment.

Harshness indicators in the ACS were not significant predictors of percentage of children aged 5–9 years. The variables that were positively associated with the percentage of children were the percentage of children between 2005 and 2009, of women who had births in the previous year, and the median number of rooms. The percentage of children and of women who had births in the previous year being significant predictors may be due to the similarity in the variables. Geographies with a higher number of children in a given year are more likely to have a higher number of children 14 years later. Another possibility is that the age range and the time difference in our data were not aligned. The middle point of our predictors using US data was 2007, and of the outcomes it was 2021. The outcome variable being children who were 5–9 years old in 2021 means that they were born between 2012 to 2016 and means that the children who were 0–4 years old in 2007 would still not be in reproductive age then. Therefore, the time span does not allow for the phenomenon described by LHT-P to be found with this outcome variable.

With all these considerations, we suggest that results found with US data were somewhat similar to the ones found with the Brazilian Census. The United States and Brazil are two of the biggest countries in land mass and both countries have population counting in the hundreds of millions. Finding similar results between the two countries is a valuable contribution to LHT-P literature. Moreover, findings that are common among different environments and that are found among a large sample size are invaluable to evolutionary psychology because of its claims of adaptations that have been selected in our evolutionary history (Buss, 2024).

Finally, we aimed at assessing whether the percentage of visible minorities, which historically have faced and still face harsher circumstances, would be a statistically significant and relevant predictor of our reproduction measures. Our results point out they were not. Having a high percentage of Black people in Brazilian municipalities had a negligible effect in predicting early reproduction and the Black or Hispanic and Latino percentages had a statistically significant negative effect in predicting higher reproduction rates in US counties. Different fields of research have long established the association between Black ethnicity in the United States and early parenthood (Wilson, 1987; Wodtke, 2013) and both Hispanic or Latino and Black women have higher fertility rates than white women in the United States (Pew Research Center, 2015). We consider that the relationship between these previous findings and the findings in our studies help in demonstrating the different circumstances and the history of racism and discrimination that visible minorities face (Bleich et al., 2019; Canizales & Vallejo, 2021; Couto & Brenck, 2024). Lower or lack of resources effects and not a general effect of ethnicity explains reproductive behavior in our model, and in the case of United States, Black and Hispanics or Latinos are actually having fewer children when the effects of such socioeconomic conditions are removed.

In sum, the usual PAT hypothesis that harshness is associated with early or more frequent reproduction was supported—at a population level—in a developing country and partially supported in a developed country. The prediction of unpredictability indicators was inconsistent in both countries, and the percentage of visible minorities were either a nonrelevant predictor or a statistically significant predictor of reproduction, but in the opposite direction as commonly observed in literature. Future research could aim to confirm such results with different data sets.

### Caveats

This research assessed hypotheses derived from LHT-P, specifically PAT. These theories are mostly used to explain the developmental trajectory and behaviors of individuals. The first study, however, used Brazilian Census data at the municipal level and the second used data at the county level. All the data utilized were the percentages of people in these geographies. Therefore, any conclusion or inference about individuals should be done with careful consideration. The first consideration is that this study relies on the assumption that most of the children from one municipality will grow up and remain in the same municipality 10 years later. If this were not true, we would be just measuring geographical correlations or some statistical artifact. The 2010 Census in Brazil reports that 62.8% of Brazilians were born in the municipality they live in (Sistema IBGE de Recuperação Automática—SIDRA, 2024) and the National Household Sample Survey indicated that in 2011 59.9% of Brazilians lived in the municipality they were born in (Goularti, 2016). The age range of most mobility is between 25 and 29 years. In comparison to the most mobile age range, the population between 15 and 19 years old was 74% as likely to move in the 2000 Census and 69% as likely to move in the 2010 Census (Nascimento et al., 2016). In addition, if the national average is around 60% of the population, it is reasonable to assume that this percentage will be negatively associated with age until the age range of most mobility. In other words, the younger the person (until 25 years of age), the more likely that she is in the municipality in which she was born. This will only not be true if one is expecting a considerable migration back to municipality of birth at a later age. We can then expect that among people 24 and younger, which is the population relevant to our outcome variable, 59.9% or more will be residing in the municipality they were born in. It is worth noting that the factor *Youth and married with children* was not a relevant predictor of *Youth Married*, an outcome factor dropped in the model. These two factors are composed of the same variables, only with the 10-year gap. This observation goes in line with the finding that *Youth and married with children* was not collinear with *Early reproduction*. All this supports our assumption that most children remain in the same municipality a decade later and supports the hypothesis that our results reveal a developmental phenomenon.

Another caveat in interpreting our findings is that the associations found at the municipal level may not be found at the individual level. It is possible that people experiencing any of our predictors (e.g., Lack of resources, Youth: Married with children, and Family size) are not the same people experiencing another predictor. It is also possible that the people between predictors and outcomes were different. In addition, there was no manipulation or random assignment of participants (characteristics of experimental research), which then increased the chances of confounding variables. Many variables that are not available in the census (e.g., mortality, disease, use of contraceptive or family planning) could be equal or better predictors of our outcome variables and can be covarying with any of our predictors. Explaining these findings then would require measurement of these other variables or another theoretical framework, which were beyond the scope of this study.

Other theoretical frameworks that could help explain the associations found in this study are, for example, genetic mediation or confounding, or cultural and institutional factors. Genetic variation may have a role in the different LHSs developed by humans (Buss, 2016; Del Giudice, 2009). It could be that genes select for or shape environmental conditions and both of these would impact LHS factors such as time of puberty (Volk, 2025).

Cultural and institutional factors could help explain the association between poverty and early parenting (Wilson, 1987; Wodtke, 2013). For example, parents who experience longer commute to work or who have to work longer hours to provide necessary income may not have as much time as parents from more affluent conditions to support or invigilate their children in their sex and reproductive decisions. In addition, poorer areas are frequently the ones that usually lack more institutions to aid parents in such tasks. Early or more frequent reproduction could be more common and more accepted in some cultures (Wilson, 1987; Wodtke, 2013). In places where educational and employment opportunities are scarce, becoming a parent may also offer a sense of identity and an “adult status” among the community (Hanna, 2001). All of these alternative explanations could interact with PAT claims to explain the association between poor communities and early and frequent parenting.

LHT-P and PAT have been subjected to many criticisms (Nettle & Frankenhuis, 2020; Sear, 2020; Stearns & Rodrigues, 2020; Volk, 2023, 2025). Critics of LHT-P and of PAT propose a considerable revision of its claims and theoretical bases. The goal of this paper was to use census data that is similar to the most common or mostly tested measures in PAT research and see if the patterns proposed by PAT would be found in this population data. This can serve as basis to theorizing about what findings are consistent in human populations and to generate more specific and formal predictions.

In sum, our findings are consistent with the idea that growing up in an unpredictable and harsh environment predicts the development of a “faster” LHS using data from the census of a developing country. A model utilizing variables measuring lack of resources, larger families, parental transition, and young parents substantially predicted a variable measuring early reproduction in both the overall sample and in the geographic areas with the highest percentages of children. However, inferences from these findings should be careful because we did not assess individual-level data.

## Conclusion

Using Brazilian Census data, we showed that harshness early in childhood in a given municipality predicts early reproduction in that municipality, consistent with the prediction of PAT. In the United States, harshness early in childhood in a particular county predicts higher reproduction rate in the same county, a finding that is similar to the findings in Brazil. However, in the United States the direction of some of the predictors was negative, which is contradictory to PAT. Ethnicity predicted reproduction in the United States but not in Brazil, suggesting that our data set cannot completely account for the effect of environmental circumstances associated with ethnicity on reproduction.

## Supplemental Material

sj-zip-1-evp-10.1177_14747049261432896 - Supplemental material for Harshness Predicts Reproduction in Brazilian Municipalities and US Counties: A Life History Theory Approach Supplemental material, sj-zip-1-evp-10.1177_14747049261432896 for Harshness Predicts Reproduction in Brazilian Municipalities and US Counties: A Life History Theory Approach by Vinícius Betzel Koehler and M. D. Rutherford in Evolutionary Psychology
